# Disparity between central and peripheral refraction inheritance in twins

**DOI:** 10.1038/s41598-021-90838-8

**Published:** 2021-06-09

**Authors:** Dibyendu Pusti, Antonio Benito, Juan J. Madrid-Valero, Juan R. Ordoñana, Pablo Artal

**Affiliations:** 1 Laboratorio de Óptica, Instituto Universitario de Investigación en Óptica y Nanofísica, Universidad de Murcia, Campus de Espinardo (Ed. 34), 30100 Murcia, Spain; 2 Departamento de Psicología de la Salud, Universidad de Alicante, Alicante, Spain; 3 Registro de Gemelos de Murcia, Departamento de Anatomía Humana y Psicobiología, Universidad de Murcia, Murcia, Spain; 4 Instituto Murciano de Investigación Biosanitaria (IMIB-Arrixaca), Universidad de Murcia, Murcia, Spain

**Keywords:** Behavioural genetics, Epidemiology, Refractive errors

## Abstract

The last decades have witnessed a sudden increase in myopia incidence among youngsters that have been related to modern lifestyle along with the use of emerging technologies affecting visual exposure. Increasing exposures to known risk factors for myopia, such as time spent indoors, close-distance work, or low-light conditions are thought to be responsible for this public health issue. In most cases, development of myopia is secondary to a vitreous chamber enlargement, although the related mechanisms and the potential interaction between central and peripheral retinal area remain unclear. For a better understanding, we performed a classical twin study where objective refractive error along 70° of horizontal retinal arc was measured in 100 twin pairs of university students, 78% of which showed manifest myopia. We found the variance of shared environmental origin (range 0.34 to 0.67) explained most of the objective refractive error variance within central 42° of the retina (22° temporal to 19° nasal), whereas additive genetic variance (range 0.34 to 0.76) was predominant in the peripheral retinal areas measured. In this sample of millennial university students, with a large prevalence of myopia, environmental exposures were mostly responsible for inter-individual variation in the retinal horizontal area surrounding the macula, while their relative weight on phenotypic variance was gradually descending, and replaced by the variance of genetic origin, towards the retinal periphery.

## Introduction

Myopia progression during childhood is caused by an enlargement of the eyeball caused by the growth of the vitreous chamber, and it is known to be linked to risk factors such as time spent indoors, close-distance work, or low-light conditions^1–8^. However, the mechanisms responsible for eye growth and the retinal area involved are still largely unknown. Over the last years, the influence of peripheral refraction (PR) in myopia development has taken the spotlight with increased research interest. However, the relationship between the visual feedback at the peripheral retina and axial elongation created a debate with opposite thoughts. The importance of peripheral optics in myopia began with the studies by Hoogerhide and Rempt et al. in 1971, where they measured peripheral refraction and predicted future myopia development in the presence of relative peripheral hyperopic defocus^9,10^. Some initial research on peripheral ocular optics supported the future myopia prediction and considered that PR with hyperopic shift could be a trigger for central myopia development^11,12^. However, several studies opposed this theory and suggested the hyperopic relative PR is perhaps a consequence of central myopia development^13–16^. Anyway, the role of different risk factors on these processes is still to be elucidated.

Twin studies allow us to estimate the relative weight of genetic and environmental factors on a given phenotype. Refractive error is no exception and several studies have estimated the heritability (i.e., the proportion of phenotypic variance due to genetic factors) of refractive error with ample variability (from 58 up to 90%) across different young and old populations^17–25^. Here we are highlighting the relevant literature estimating foveal refraction inheritance from the last two decades. Niels Lyhne et al.^21^ found 90% heritability among a Danish population (age 20 to 45 years) and Dirani et al.^23^ found high heritability in an Australian sample (age 18 to 88 years) showing 88% heritability in men and 75% in women. A sample from the British population also showed high heritability in a study conducted by Hammond et al.^22^ (sample age 49 to 79 years) showed heritability of 84%. A comprehensive table of all relevant studies done from 1962 has been provided in our previous work^26^. Most of these studies were conducted either across a general population including all age groups or an old population. Furthermore, none of them captures the environmental changes that have occurred during the last two decades which was evident in a recent work, where heritability of objective refraction in the sample used for the present study was considerably lower compared to a middle-aged twin population from the same geographical origin^26^. Sample characteristics, such as age, years of schooling or geographical area may be responsible for those inconsistencies. Moreover, most previous heritability studies were restricted to estimate the variance of on-axis refraction, showing a general trend of substantial heritability, while there is a scarcity of studies exploring the genetic and environmental influences on PR. There is only one published study estimating PR heritability, conducted in Chinese children and adolescents, which reported a significant role of additive genetic factors to explain PR variance^27^, thus supporting a relevant responsibility of genetic factors on the refractive error variance. However, this study included a wide age span, ranging from children to young adults (8–20 years), with very different levels of physical development and visual need or exposure, and it was limited in that they only studied PR at a single peripheral angle (40° from the fovea); neither did it provide information about the refractive error at the line of sight (central measurements). This approach, using a single point estimate, may not be useful for identifying trends or change patterns, it could miss relevant shifts in refraction across the retina, and it could be more susceptible to possible measurement errors than multi-point approaches, resulting, in general, in a less sensitive measure. Hence, we found a gap in the literature regarding possible influence of heritability at different retinal locations in the periphery.

A classical twin study was designed to extend our understanding of the developmental underpinnings of objective peripheral refraction variance in a sample of millennials (i.e., subjects born at the end of the twentieth century); a generation that is known to have suffered a large increase in the incidence of myopia in developed countries. In order to do that, we have performed a series of univariate twin models, covering a wide retinal area, to estimate the relative weight of genetic/environmental factors in the variability of objective refractive error along a 70° retinal horizontal arc. We used wave-front sensing technology to measure PR at wide retinal eccentricities with high resolution (every 1-degree interval). The point-by-point refraction data gave us the power to obtain a detailed estimation of the genetic and environmental influences on PR across a large retinal area to offer information about the impact of environmental exposures on the mechanisms of eye development.

## Results

This study included 200 participants (100 twin pairs) with an average age of 22.6 ± 4.0 (range 19 to 30) years and 21.4 ± 2.4 (range 19 to 36) years among MZ and DZ twin pairs, respectively. Average central objective refraction (spherical equivalent; SE) was − 2.08 ± 2.17 D (range + 3.8 to − 7.0 D) in MZ twins, and − 2.28 ± 1.87 D (range 0.0 to − 9.8 D) in DZ twins. Participants showed a significantly higher female participation in both groups: 83% in MZ and 69% in DZ. However, the mean effects of age and sex were added to the model as covariates to control for their effect^28^. Study participants had a considerable high prevalence of myopia, where 78% showed myopia (SE < − 0.50 D).

Twin correlations were always higher for MZ (range 0.785–0.917) than for DZ (range 0.355–0.695) across all retinal eccentricities, indicating a substantial role of genetic factors across all retinal eccentricities (Supplementary Table 1). Although for some of the data points, the correlation patterns (rMZ > 2rDZ) suggested the possibility of non-additive genetic effects, full ACE models were run for all retinal eccentricities in order to keep meaningful comparisons across all measures.

**Figure 1:**
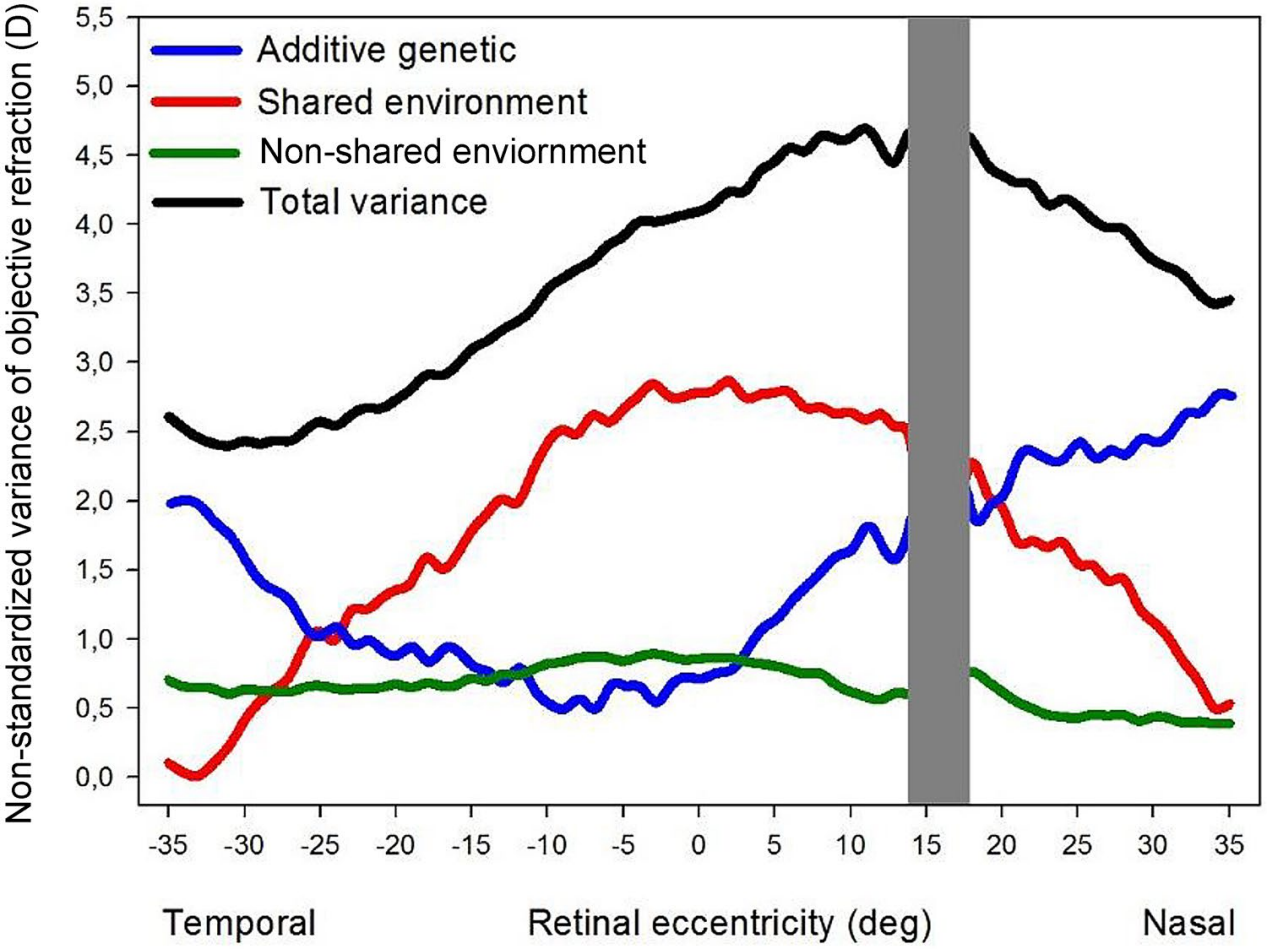
Variance components (ACE model) of objective refractive error, estimated by SEM analyses, across the horizontal retina. 0° represent the position corresponding to the fovea; negative values indicate temporal retina, and positive values indicate nasal retina. Lines represent non-standardized estimates of additive genetic (blue), shared environmental (red), non-shared environmental (green), and total phenotypic (black) variance. The Grey area represents the area affected by the optic disk.

Figure 1 shows non-standardized estimates of corresponding genetic, environmental and total variances at 71 retinal eccentric points (± 35°). The black line illustrates total phenotypic variance, while blue, red and green lines denote additive genetic, shared and non-shared environmental variances respectively. We found an average total variance of 3.64 ± 0.76 D with peak value at the central retina (4.69 D at 11° nasal retina) and gradual fall towards the periphery. The individual average phenotypic components were 1.38 ± 0.66 D, 1.64 ± 0.81 D, and 0.62 ± 0.14 D for additive genetic, shared and non-shared environmental variances respectively. The additive genetic and shared environmental variances showed opposite trends across analyzed retinal eccentricities (blue and red lines respectively). While additive genetic variance reached its minimum (0.46 D) at 7° nasal, showing a gradual rise towards both sides, shared environmental variance showed an opposite trend, reaching its maximum (2.70 D) at 2° nasal and decreasing thereafter. The non-shared environmental variance showed no significant fluctuation across the horizontal retinal eccentricity.

**Figure 2:**
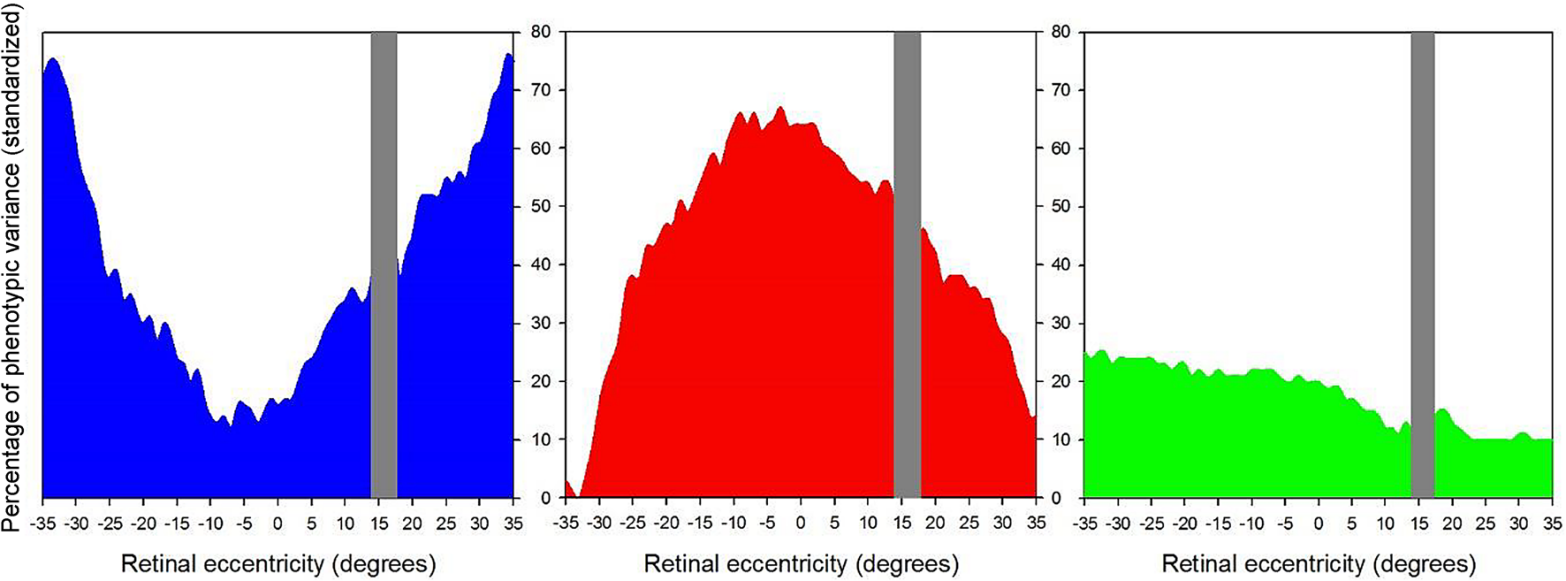
Percentage of phenotypical variance for variance components (ACE model) of objective refractive error, estimated by SEM analyses across the horizontal retina. 0° represent the position of the fovea; negative values indicate temporal retina, and positive values indicate nasal retina. Left: heritability (blue). Center: shared environment (red). Right: non-shared environment (green). The Grey area represents the area affected by the optic disk.

Standardized estimates were calculated as the proportion of phenotypic variance explained by additive genetic (A—heritability), shared (C) and non-shared (E) environmental variances respectively. Figure 2 represents the proportions of total phenotypic variance accounted for by additive genetics (A; Fig. 2, left) shared environment (C; Fig. 2, center), and non-shared environment (E; Fig. 2; right) at each angle of horizontal eccentricity from 35° nasal to 35° temporal retinal eccentricity.

We found an average heritability of 39 ± 19% (calculated as the sum of all h^2^ values for each retinal eccentricity and divided by the total number of estimations) having a large variability depending on horizontal retinal eccentricity (Fig. 2, left). Additive genetic variance accounted for a minimum of 12% and a maximum 76% of the phenotypic variance which indicates that the genetic and environmental influences are different depending on the retinal eccentricity point. The lowest values were around the fovea, while the highest was observed at distal points of maximum eccentricity (temporal and nasal). Following this ample variation, the data from the areas beyond 22° in temporal retina and beyond 19° in nasal eccentricity were best fitted with a constrained AE model (Supplementary Table 2), where the shared environmental component could be dropped without deterioration of model fit. On the contrary, a CE model, where the genetic component could be dropped, showed the best fit in most of the central retinal zone (Supplementary Table 2).

## Discussion

We performed a thorough point-by-point analysis to reveal any refraction heritability shift across a wide range of the horizontal retinal scan. With the comprehensive SEM analyses, we found the highest total phenotypic variance (black line in Fig. 1) concentrated around the central retina, which gradually decreased towards the periphery. The contribution of genetic and environmental components to phenotypic (i.e., peripheral refraction) variance fluctuated at different retinal areas. The additive genetic influence was lowest at the foveal region, where the influence of shared environmental factors was highest. Interestingly, the decreasing total phenotypic variance towards both sides of the peripheral retina coincided with increasing additive genetic variance (Fig. 2, left) accompanied by a gradual decrease in shared environmental variance (Fig. 2, center).

The gradual increment of heritability towards the peripheral retina was distributed in such a manner that beyond specific thresholds (19° nasal and 22° temporal), it provoked a shift in the best fitting model from an environmental (CE) to a gene-environmental combined (AE) model. This deviation and the low heritability at the area surrounding the fovea suggest that variability at the central retina (around ± 20° from the fovea) is greatly influenced by environmental factors than variability at the more eccentric areas of the retina. Moreover, the lower magnitude of unstandardized genetic variance around fovea would also indicate less dependence on genetic factors in this area, suggesting a higher sensitivity to the variation produced from environmental inputs. Consequently, our results would suggest that differences in myopia development are mostly explained by environmental effects that influence the eyeball at the central retinal zone. In contrast, eccentric zones of the eyeball would be less sensitive to environmental effects and dependent on genetic architecture. Concurrently, it is important to stress that the total variance and the raw shared environmental variance (Fig. 1) were both highest at the central retina, suggesting greater visual exposure at central retinal region than at peripheral areas. Considering the above-discussed findings and the fact that the non-shared environmental variance remains stable across the analyzed retinal eccentricity, such environmental influences should be looked for within the shared visual exposures among siblings.

In agreement with our peripheral refraction heritability result, the study by Ding et al. in children and adolescents also reported a significant role of the additive genetic component to explain the variance of peripheral refraction^27^. However, they did not provide information about central and mid-peripheral refraction heritability. We couldn’t find any other published literature on peripheral refraction inheritance. Whereas, all other studies on refraction heritability were restricted to foveal refraction only and generally suggesting a strong genetic influence on its variance^17–25^. On the contrary, the foveal and mid-peripheral refraction heritability in our study population showed a lower foveal refraction heritability with increased shared environmental impact.

The higher shared visual exposures at the central retina and high myopia prevalence (78%) in our study population, may most likely include myopigenic factors: prolonged near visual tasks, time spent indoors, lighting conditions, and reading text contrast or polarity etc. These factors are well connected to the modern lifestyle, massive urbanization and constant use of display terminals, as suggested by recent myopia studies^5,6,29–31^. Our study population was also exposed to all these myopia causative factors as they are university students and belong to an urban area of a developed country. Myopia theories like ‘accommodation lag theory’ and ‘mechanical tension theory’ can further explain the mechanism of myopia development in a myopigenic environment prioritizing prolonged near-work^32–34^. Development of myopic refractive error is mainly related to an increase in axial length (AL), following a rule-of-thumb that every 300 microns AL growth causes one diopter rise in myopia, considering no alteration of other ocular components^35,36^. However, the AL growth is unlikely to always be confined at the macular area but extends to an unknown area at the posterior ocular wall. Based on biometrical and optical measures, Atchison et al.^37^ proposed three possible scenarios to explain ocular growth linked to myopia: global expansion of the posterior chamber, extension of the posterior chamber induced by equatorial stretching of the eyeball, and posterior pole theory with an ocular growth confined to the area surrounding the macula. However, it has been complicated to test these theories so far due to the limitations of the methods used for retinal off-axis measures. We used an alternative approach by analyzing a wide range of off-axis refraction in twin siblings, who share genetic inheritance and environmental exposures. Thenceforth, we computed the relative influence of individual phenotypic components on its variance of refraction. An extensive SEM analysis of peripheral refraction applied in this sample allowed us to differentiate to which point the variance of refractive error, measured from the line of sight up to ± 35° retinal area, was influenced by the variance of genetic origin or visual exposures. The central large environmental influence found in our study participants may predict a trend of ocular axial length growth limited mainly at the posterior pole of the eyeball, in an area from the optical disk to around ± 20°, thus supporting the ‘posterior pole elongation’ myopia theory.

We could further connect these results with the neural and optical limitations of the retina resulting in poor sampling resolution at the periphery. The central retina is more sensitive to visual exposures (environmental influences) than the periphery mostly due to the high retinal resolution sensitivity threshold in the fovea, which rapidly decreases towards the peripheral retina^38^. Peripheral vision is compromised not only because of low cone density and limited ganglion cell density^39^, but also for increased optical aberrations at the peripheral retina^16,40,41^.

Although myopia is not a disease itself, high myopia is a risk factor for severe ocular diseases and even blindness^42–48^. This study may contribute to facing this challenge, as it provides evidence and suggests mechanisms for environmentally driven myopia development. The obtained results from our study sample may represent a large part of the Spanish young population as a recent survey in 2019 showed higher education rate in whole Spain was 51% beyond the age of 20 years^49^. Whereas, Murcia region alone showed a 47% higher education rate for the academic year 2016–17^49^. This study also has some limitations that need to be taken into account. Among them, a larger sample would have allowed for specific group analyses (i.e., different refraction types, sex, or age groups). Additionally, it was not possible to collect extensive information about environmental exposures that could have enriched our analyses and conclusions.

In summary, the refractive error variance showed a shift in the relative influence of genetic and environmental factors across horizontal retinal eccentricities. The total phenotypic variance showed its highest concentration at the macular region with gradual descent towards the periphery. Heritability showed an opposite pattern with its highest at the peripheral retina with gradual depletion towards the fovea. We found the influence of shared-environmental factors to be the main source of individual differences explaining the central peak of total phenotypic variance.

## Methods

### Study population

Peripheral refraction was measured in 100 twin pairs (54 MZ and 46 DZ), from the Murcia Twin Registry (MTR)^50,51^. All study subjects were either alumni or pursuing university students at Southeast Spain with an age range of 18 to 30 years in MZ and 18 to 36 years in DZ twins. Zygosity of the twins was confirmed by DNA analysis for same-gender twins. The study was designed considering the tenets of the Declaration of Helsinki49 and informed consent was obtained from all study participants. The ethical approval was granted by the Research Ethics Committee of the University of Murcia (ID: 1108/2015). Subjects were recruited considering specific exclusion criteria, which includes active ocular pathology or allergy, previous ocular surgery, use of rigid contact lenses as could be in Ortho-k treatments, ocular trauma, amblyopia or a decimal corrected distance visual acuity below 0.9. The right eye measurements were considered in all cases as we found a very high correlation between both eyes. The present study sample was used previously to access heritability at fovea^52^.

### Peripheral refraction

Peripheral wavefront aberrations were measured using an open-view Hartmann-Shack (HS) sensor (VPR, Voptica SL, Murcia, Spain). The VPR can scan a wide range of horizontal arc of 80° (± 40°) and provides 81 high-resolution HS images (measurements for each 1-degree interval) in 1.8 s. A complete measurement consists of four consecutive scans, resulting in 324 high-resolution HS images. Since often the data recorded in the extremes can be noisy, we decided to include only data for 70° (± 35°) scan arc. A near-infrared laser light source (780 nm) was used in the instrument considering ocular conform with a minimal pupillary light response. The intensity level (10 µW) was kept much lower than the permissible ocular safety limit standard for the selected wavelength. Further detail about the peripheral wavefront sensor can be found elsewhere^53^.

During measurement, subjects were asked to fixate at a laser fixation point at 3 m distance while keeping a close eye on measurement fluctuations to avoid accommodation. The obtained HS images were then processed with a processing software (Voptica SL, Murcia, Spain) for 4 mm pupil and 5th order Zernike polynomials. Spherical equivalent (sphere + ½ cylinder) value was used as objective refraction at each retinal eccentric point.

### Data analysis

Statistical analyses were performed using SPSS 24.0 (SPSS Inc. Chicago, IL) software and the OpenMx package in R^54^. Normal distribution was checked by means of the Kolmogorov–Smirnov test. Differences between variables were obtained employing the Student t-test for normally distributed and the U Mann–Whitney test for non-normally distributed variables. The intraclass correlation coefficient (ICC) was used instead of the Pearson correlation coefficient to avoid problems with twin data dependence while performing the comparison between siblings. To estimate the components of phenotypical variance the data were analyzed using Structural Equation Modelling (SEM). SEM allows for a decomposition of the phenotypic variance into additive genetic factors (A; the sum of allelic effects across all loci); non-additive genetic effects (D; the effects of genetic dominance and, possibly, epistasis); shared-environmental (C; environmental influences that make family members more alike) and non-shared or individual factors (E; idiosyncratic and random environmental influences that make family members less alike. It also includes measurement error). The classical twin study makes it possible to make use of the difference between MZ twins who share 100% of their DNA and DZ twins who share on average 50% of their segregating DNA. C and D cannot be estimated at the same time in a classical twin study using only data from twins reared together. Hence the selection of a model including ACE or ADE components is based on the pattern of twin correlations. An ADE model is usually selected when the DZ correlation is lower than half of the MZ correlation. In contrast, an ACE model is selected if the DZ correlation is greater than half of the MZ twin correlation^55^. Mean effects of age and sex were added to the model as covariates to control their effect^56^.

### Univariate analyses

One univariate ACE model was fitted to the data for each one of the 71 measured degrees. The ACE model was used for all the measured points in order to keep meaningful comparisons across all measures. The ACE model is also considered the standard approach to this kind of analysis. Assumptions of twin modeling were checked in the saturated models.

Heritability is defined as the proportion of the phenotypic variance explained by genetic factors (the same applies to common and non-shared environmental factors)^31^. Unlike other publications, where unstandardized variance components are not usually of interest, raw or unstandardized variance changes across each measured degree are reported. This allows us to compare the actual magnitude of the variance produced by genetic and environmental influences across all the retina points and not just their relative proportions, which could yield similar estimates at every point if the magnitude of the different sources of variance grow or decrease in parallel. Hence, both raw and standardized variance components are presented (Figs. 1, 2 and Supplementary Table 2).

Nested sub-models (i.e. AE, CE and E) were also fitted to check if any of the components (A, C, or AC) could be dropped without a significant worsening of the model fit. The fit of each model was tested using the likelihood-ratio chi-square test and the Akaike’s information criterion (AIC)^56^ (Supplementary Table 2).

## Supplementary information

Supplementary Information 1.

Supplementary Information 2.

## Data Availability

Raw data, documentation, and code used in analysis (excluding any personal info) will be available upon request.
